# A Field-Based Screening Protocol for Hamstring Injury Risk in Football Players: Evaluating Its Functionality Using Exploratory Factor Analysis

**DOI:** 10.3390/sports13090295

**Published:** 2025-09-01

**Authors:** Nikolaos I. Liveris, Charis Tsarbou, George Papageorgiou, Elias Tsepis, Konstantinos Fousekis, Sofia A. Xergia

**Affiliations:** 1 Department of Physiotherapy, School of Health Rehabilitation Sciences, University of Patras, 26504 Rio, Greece; ctsarmpou@upatras.gr (C.T.); tsepis@upatras.gr (E.T.); kfousekis@upatras.gr (K.F.); sxergia@upatras.gr (S.A.X.); 2 SYSTEMA Research Centre, European University Cyprus, P.O. Box 22006, 1516 Nicosia, Cyprus; g.papageorgiou@euc.ac.cy

**Keywords:** injury prevention, athletes’ screening, risk factors assessment, field-based testing

## Abstract

This paper propose a practical field-based screening protocol for evaluating the risk of hamstring injury. This is done by discerning the most important factors that better explain the underlying structure among various measurements. Following a cross-sectional study design, ninety-nine professional and semi-professional football players were assessed at the team’s facilities during the preseason period. The collected data included aspects of demographic characteristics; previous injuries; athlete sense of burnout (Athlete Burnout Questionnaire (ABQ)); hamstring (HS) flexibility (passive single leg raise test); isometric hamstring strength (make and brake test); isometric quadriceps strength; single-leg triple hop for distance; endurance of the core muscles (prone bridge, side bridge and Biering–Sørensen tests); and hamstring strength endurance (single leg hamstring bridge test). Subsequently, Exploratory Factor Analysis was performed. Following a summarized dimension reduction process, the twenty-three assessment variables were grouped into a parsimonious model of six main risk factors. Specifically, the resulting model explains 55.7% of the total variance, comprising HS and core endurance (20.2% of the variance), HS strength (12.8%), previous injuries (8.9%), ABQ (5.8%), lower limb strength (4.1%), and strength limb symmetry (3.8%). The proposed model provides a practical protocol, facilitating sports scientists in evaluating the risk for HI in the highly complex reality of field-based situations.

## 1. Introduction

Hamstring injuries (HI) are associated with substantial absence from sports and elevated re-injury rates, leading to significant implications for athletes and financial problems for teams [1,2]. Despite considerable research, HI rates continue to increase, constituting up to 24% of all injuries in professional football [1]. The incidence of HI is, on average, 0.8 per 1000 h of exposure, and there is a 13% prevalence of HI during nine months of exposure [3,4]. In addition, HI is more likely to occur during matches than in training [3,4]. Regarding the affected muscles, previous investigations have proved that the biceps femoris represent 79% of all HIs [5].

The mechanism of HI includes high-speed and accelerated athletic activities that place the hamstring muscles under high eccentric demands [5]. The movement patterns that have been associated with HI include running-related activities, in which hamstrings are more likely to be injured during the late swing phase of high-speed running. In addition, sporting activities such as lunging, tackling, or kicking have been linked with the HI mechanism [5]. Football is among the team sports with the highest HI rates, as it requires athletic activities that expose hamstrings to high loads, increasing the risk for injury [4,6].

Multiple risk factors have been identified [7], with prior HI and advancing age being most prominent. In addition, factors related to workload, such as week-to-week changes in high-speed running exposure, seem to be of great importance [8,9]. Further, despite conflicting evidence, deficits in core stability during high-speed running [10,11], decreased hamstring strength, decreased ballistic ability, lower hamstring endurance, and hamstring strength asymmetries are among the important internal risk factors. Finally, there is evidence that psychological factors, such as an increase in the sense of burnout, may affect the risk of HI [12].

Given the wide range of risk factors implicated in the risk of injury, previous studies have shown that HI is a complex and dynamic phenomenon where risk factors interact non-linearly, leading to a plausible injury [7,13]. Additionally, it is suggested that athletes’ internal risk states change throughout the season, underscoring the need for ongoing assessments to more accurately determine when an athlete faces an increased risk of injury [13,14]. Preseason risk factor screening is important to determine an athlete’s condition at the initial state of the season and plan initial preventive interventions, but it is impossible to capture the condition of an athlete during the season, which is when most injuries occur [13,14]. Athletes’ injury risk state cannot be considered as a steady state, which is the hypothesis of most preseason evaluations reported in the literature, but rather a dynamic condition that changes rapidly throughout the season [14,15]. From that point of view, preseason examinations have limited injury predictive ability and represent one of the main limitations of the prospective studies that evaluate the predictability of various measures [13,14]. During the season, athletes’ conditions change rapidly, due to the effects of training, preventive interventions, workload, fatigue, and psychological factors [13,14,15]. Consequently, rapid in-season evaluation can offer more valuable insights into an athlete’s injury risk and improve the accuracy of injury prediction. It also enables a more timely and precise implementation of injury prevention interventions [13,14]. However, most reported measures require laboratory assessments, such as isokinetic testing and three-dimensional kinematics analysis, making the application of such measures for football teams’ staff a significant challenge [7].

Consequently, a need arises for a practical, structured protocol so that sports scientists can effectively employ it in field-based situations, which can be efficiently repeated during the season to monitor possible modifications of the athlete’s risk profile [15]. This could enable individualized adjustment of exercise-based preventive interventions and identify athletes who are at increased risk of injury. For instance, a significant decline in hamstring strength, increased strength asymmetries, or an increased sense of burnout among consecutive measures may indicate increased fatigue. Thus, specific interventions should be applied to this athlete, such as reducing training loads and altering exercise-based preventive interventions [15].

This paper aims to propose such a protocol by discerning the most important underlying factors that better explain the structure among measured variables obtained from a field-based screening. This is accomplished by assessing the interdependence of the measured variables using Exploratory Factor Analysis (EFA). As a result, we propose a field-based screening protocol that provides essential information to evaluate HI risk using the most practical, easy-to-apply measurements with the use of portable, low-cost equipment.

## 2. Materials and Methods

### 2.1. Study Design

A cross-sectional study was conducted, wherein athletes were assessed during the initial two weeks of the 2022 preseason phase. The screening protocol was employed in the teams’ facilities using questionnaires and clinical tests to collect data regarding previous injury characteristics, sense of burnout, lower limb strength, core and hamstring muscle endurance ability, and hamstring flexibility. This investigation is part of a more extensive study registered in the public database of ClinicalTrials.gov (ID: NCT05425303). The study has obtained approval from the Institutional Ethics Committee of the University of Patras. The Strengthening the Reporting of Observational Studies in Epidemiology (STPOBE) guidelines [16] for cross-sectional studies were utilized for reporting this study.

### 2.2. Participants

A total sample of ninety-nine football players (Mean, standard deviation; SD), of age 22.2 (5) years, weight 74.4 (7.7) kg, height 179.8 (6.3) cm, and BMI 23.2 (1.8), was recruited for this study. This sample included 52 professional football players for three teams participating in the second Greek division (age 22.5 [4.5] years, weight 75.4 [7.8] kg, height 179.9 [6.6] cm, BMI 23.3 [1.7] and 47 semi-professional football players from one team participated in the third Greek division, and one in the under−19 Greek division (age 21.8 [5.5] years, weight 73.3 [7.6] kg, height 177.6 [5.7] cm, BMI 23.2 [1.8]). The participants represent a convenience sample, selected based on the teams’ positive responses to the invitation to participate in the study. Among the male football players considered for the examination (*n* = 125), ninety-nine ultimately participated in the pre-screening process. Twenty-six players were excluded due to recent and not fully rehabilitated injuries, scheduling restrictions set by their teams, or incomplete measurements caused by pain or instability. Athletes included in the study were free of injury, had professional contact with their teams, and had taken part in five to six training sessions per week the previous season. Table 1 provides the main characteristics of all football players who participated in the study.

### 2.3. Measurements

Firstly, participants were informed about the scope of the study, then read and signed the information and consent form. Then, participants filled out three main self-reported questionnaires. The first questionnaire included details about demographic characteristics and details regarding football participation, such as years playing at the professional level, hours trained the previous year, days trained per week the previous year, and games participated in over the previous year. In addition, a second questionnaire was formulated based on previously reported guidelines [16] to record details about previous injuries. Particularly, the collected previous injury parameters included the number of previous injuries, the type of injury, the injured lower limb, the year and month of injury, and time loss due to injury recorded on a 5-point scale (0 = no time loss; 1 = less than 8 days; 2 = 8–28 days; 3 = 1–3 months; and 4 = greater than 3 months). Thirdly, the Greek version of the Athlete Burnout Questionnaire (ABQ) [17] was used to collect details regarding the athletes’ sense of burnout. The ABQ has been previously translated, validated, and evaluated for its reliability in a Greek athlete population study by Markati et al. [17]. The Greek adaptation of the ABQ consists of 13 items addressing three dimensions of athlete burnout. These dimensions include “physical–emotional exhaustion”, assessed through five items; “reduced sense of accomplishment”, also assessed through five items; and “sport devaluation”, measured with three items. It is a self-reported questionnaire, and responses were given on a 5-point Likert scale, ranging from almost never (1) to almost always (5). Athletes completed all questionnaires with the assistance of an examiner.

**Figure 1:**
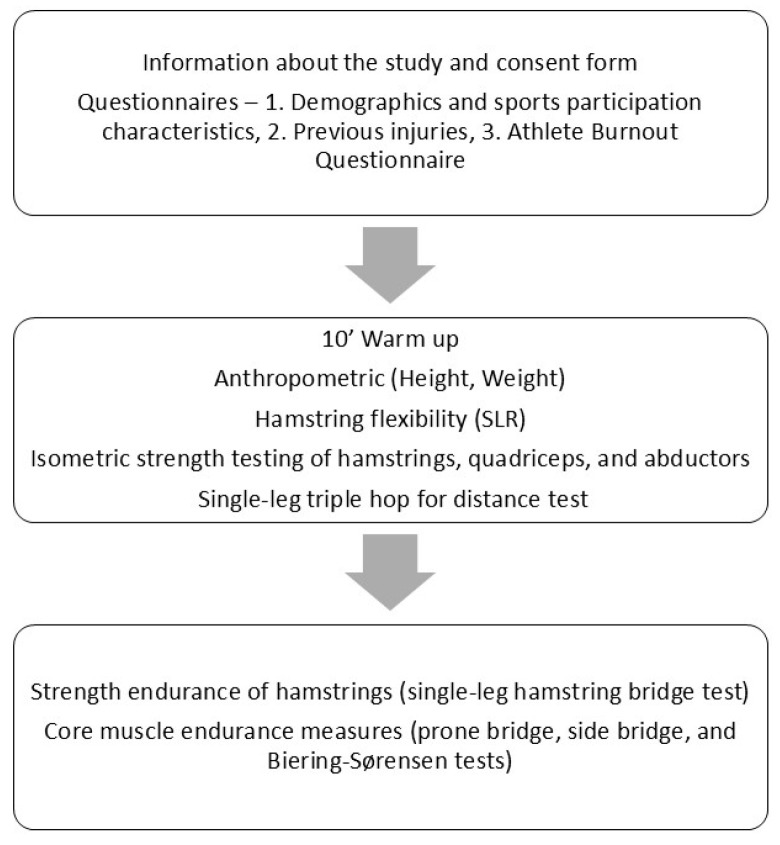
A graphical representation of the data collection process.

After completing the questionnaires, athletes performed a ten minute sports-specific warm-up that included running, stretching, and agility exercises. Then, height was measured, and athletes took part in the evaluation measurements. The measurement of weight was conducted using a force platform (Bertec, Columbus, OH, USA), which was employed to assess athletes’ landing mechanisms as part of a concurrent study [18]. The neuromuscular evaluation protocol included measures of hamstring flexibility; isometric strength of hamstrings, quadriceps, and hip abductors; the single-leg Triple Hop for Distance (THD) test; strength endurance of hamstrings; and core muscle endurance measures, including rectus abdominis, lateral abdominis, and back muscles. The above measurements were applied in the order mentioned above, with approximately five minutes’ rest between the measures to reduce the possible effect of fatigue. Three examiners conducted all the measurements, with each of them focusing on a specific part of the protocol. More precisely, the first examiner was responsible for initially informing the participants, administering questionnaires, and collecting the anthropometric variables. The second examiner carried out flexibility and isometric strength measures. The third examiner was responsible for the application of the THD test, as well as hamstrings and core muscles endurance measures. All examiners were physical therapists: two PhD students and one MSc student with 5–10 years of clinical experience. Before the study, pilot applications were conducted to test the application of the entire protocol and to familiarize the examiners with the measurements. More specific details about the tests used and the measurement configuration are explained below. In addition, Figure 1 presents a graphical representation of the measuring protocol.

Regarding the specific measurements that were applied, initially, hamstring flexibility was evaluated using the passive Straight Leg Raise (SLR) test [19]. During this test, the athlete lay supine as the examiner flexed the hip with the knee extended. Hip flexion degrees were measured using a bubble inclinometer (Baseline, New York, USA) positioned at the lower third of the tibia. The measurement was taken during hip flexion, with tension applied to the posterior kinetic chain just before pelvic movement was observed [19]. The average value of the two measures was used for the statistical analysis. A third measure was performed when there was a large difference between the two measures (>5%) [19].

Afterwards, athletes were subjected to an isometric strength evaluation of their hamstrings, quadriceps, and abductors muscles using a handheld dynamometer (HHD) (MicroFET 2; Hoggan Scientific, Salt Lake City, UT, USA). The use of HHD has proven to be an adequate option for field-based evaluation, providing moderate to good validity, reliability, and correlation with the isokinetic dynamometer [20,21]. Before the data collection, athletes performed two submaximal muscle contractions to familiarize themselves with the strength tests. Subsequently, three maximal voluntary contractions lasting approximately five seconds were performed, and the highest value was used for the statistical analysis. Measurements were recorded in Newtons (N) and subsequently multiplied by the lower limb length for abductors assessment and by tibia length for quadriceps and hamstrings evaluation, resulting in values expressed as Newton-meters (Nm). These values were then normalized by dividing by the athlete’s body mass in kilograms (kg). A normative value, expressed as Nm/kg, was utilized for statistical analysis. During the execution of muscle strength testing, athletes were stabilized by one of the examiners and using a stabilization belt. All isometric strength measures were performed by the same examiner, in the following order of hamstring “make test”, hamstring “brake test”, abductors, and quadriceps. To minimize fatigue from the maximum voluntary contraction, a 20 s rest was applied between the three measurements of each muscle group, and a 1–2 min rest was applied between the tests of the different muscles.

Particularly, hamstring isometric strength was evaluated using the “make” and “brake” tests [21,22]. Both tests were performed with the subject in a prone position and their feet extending beyond the end of the examination table. The tests were performed with the knee flexed at approximately 30° and the HHD positioned at the athlete’s heel. In the case of the ‘make” test, athletes were informed to push forcefully against the dynamometer for approximately five seconds. For the “brake” test, athletes were initially asked to perform an isometric contraction against the dynamometer, and after approximately three seconds, when the examiner ensured that the peak isometric contraction had been achieved, the examiner broke the knee angle by pushing the knee to extension.

Quadriceps isometric strength was measured with the athletes in a sitting position and their backs upright, upper limbs crossed on their chest, and a 90 degree flexion angle at hip and knee joints. The athlete forcefully extended the knee against a stabilized belt. To prevent possible pelvic and thigh motion during the maximal isometric contraction, one of the examiners stabilized the athlete’s pelvis, with his hands positioned near to anterior superior iliac spine. This test setup has been proven to provide an acceptable level of validity and reliability for an isometric strength assessment of knee extensors [23].

The isometric strength of the hip abductors was evaluated, following previously published instructions [24]. This evaluation protocol has been proven to have high to excellent reliability, providing ICC values ranging from 0.76 to 0.98, and is supposed to provide adequate stabilization of the pelvis during the isometric contraction. The participant was lying in a side position with the testing lower limb on the upper side, with the hip joint in a neutral position and the knee in extension. The contralateral lower limb was flexed at the hip and knee, while the subject held the examination table. The examiner was behind the athlete, placing the HHD 5 cm above the lateral malleolus with one hand and supporting his pelvis with the other hand. Subsequently, the athlete pushed forcefully against the HHD for five seconds to obtain a maximum isometric contraction.

After the strength measurements, the hop distance was measured using the single-leg Triple Hop for Distance (THD) test. THD has been proven to have high test re-test reliability, and constitutes a functional measure of lower limb power and strength [25,26]. After two familiarization attempts, three successful efforts at each lower limb were recorded. Athletes must maintain balance for 2 s when landing to record a successful trial. The test was performed alternatively for each lower limb, separated for 20 s rest between the trials to prevent fatigue. The highest value recorded in centimeters and divided by the athlete’s height as a normalized value was used for the statistical analysis [25,27].

Finally, four tests measuring the endurance of hamstrings and core muscles were applied. To avoid fatigue influencing other measurements, strength endurance tests were performed at the end of the screening protocol. Participants were given an adequate rest period of about 3–5 minutes following the other assessments. To measure the endurance of hamstring muscles, the Single Leg Hamstring Bridge (SLHB), as previously reported [28], was performed. The participants were positioned supine with their tested leg’s heel resting on a 60 cm platform, maintaining approximately 20° of knee flexion. The contralateral leg was stabilized in a flexed position at both the hip and knee, while the participants’ arms were folded across their chests. To complete the task, participants were required to elevate their pelvis until they achieved 0° hip extension, briefly touched the ground, and then immediately lifted their pelvis back to 0° hip extension without pausing. After a familiarization trial, athletes executed as many repetitions as possible until exhaustion. The examiner cautiously evaluated the accurate execution of the test, giving motivation to athletes. In the case of an incorrect technique, one warning was given by the examiner and the test was terminated in the next error [28]. The maximum repetitions were recorded for further analysis.

In addition, three specific measurements were utilized in a random sequence to evaluate core stability and muscle endurance. The prone bridge test was utilized to evaluate rectus abdominal muscle endurance, as previously reported [29]. The test has proven acceptable reliability and validity [29]. The lateral abdominal muscles’ endurance ability was evaluated bilaterally using the side-bridge test following the proposed guidelines [30]. Finally, the endurance ability of the core extensor muscles was evaluated by utilizing the Biering–Sørensen test [30,31]. This test has been proven to have acceptable validity for this purpose [31]. The examiners informed the participants about the accurate execution of core muscle endurance tests, and the athletes performed a familiarization trial. In all core endurance measures, athletes should keep the correct position until exhaustion. The tests were terminated in any case of discomfort and if participants failed to maintain the right position for two seconds. The examiner motivated athletes to persist in an accurate position. The hold duration was measured from when the athlete assumed the testing position until they could no longer maintain it due to exhaustion. The maximum time was used for statistical analysis [29,30,31].

### 2.4. Data Processing and Statistical Analysis

Initially, all data was recorded in Microsoft Excel. Interlimb symmetry was calculated for all bilateral strength and flexibility measures using the formula [(dominant-nondominant)/maximum (dominant or non-dominant) *100] [32]. Regarding the previous injury data, the number of all previous injuries for each athlete for both lower limbs and the time loss of the most recent injury recorded in a 5-linked scale (0 = no time loss; 1 = less than 8 days; 2 = 8–28 days; 3 = 1–3 months; and 4 = greater than 3 months) were used for statistical analysis. In addition, the three dimensions of the ABQ (Emotional Physical Exhaustion, Reduced Sense of Accomplishment, Devaluation) recorded in a 5-linked scale were included in the analysis. The neuromuscular measures inserted in the statistical analysis included SLR recorded in angles; isometric strength peak values of the hamstrings, quadriceps, and abductors recorded in Nm/kg; maximum distance in THD test; maximum repetitions in SLHB; and the maximum time in prone bridge, side bridge, and Biering–Sørensen tests. For bilateral measurements of lower limb strength, SLR, THD, and SLHB, only the data for the dominant limb were used in the statistical analysis. In total, twenty-three parameters were analyzed.

Subsequently, the data were entered into SPSS v.28 for statistical analysis. The initial step involved screening for missing values, outliers, multicollinearity, and normality. Normality was evaluated using the values of skewness and kurtosis, and visually with histograms. Skewness values up to 2.0 and kurtosis up to 7.0 are considered acceptable thresholds [33]. After initial data screening, descriptive statistics were calculated for all data using the mean and standard deviation. The correlation matrix was also assessed to detect correlations among measurement items.

Then, Exploratory Factor Analysis (EFA) was performed to identify the best factor solution that better interprets the underlying structure of the measured variables based on their associations [33]. EFA is a powerful multivariate statistical method that analyzes the associations of multiple variables simultaneously and proposes a grouping of these variables into factors based on the variables’ interdependences [33]. When assessing data suitability for EFA, it is essential for Bartlett’s test of sphericity statistics to be significant (*p* < 0.05). Additionally, while Kaiser–Meyer–Olkin (KMO) values above 0.70 are preferred, values below 0.50 are deemed unacceptable [33]. The Promax rotation and the Principal Axis Factoring extraction methods were used as more accurate methods that better represent reality, in contrast with softer solutions like Varimax rotation and principal component analysis [33]. Then, the measurement items’ commonalities were assessed. Measurement items showing low communality values (<0.30) are potential candidates for exclusion from the EFA after considering the pattern matrix results. Low communality values indicate that a variable has a low correlation with the other variables and possibly results in low loadings in various factors in the EFA results (cross-loading). To decide the number of factors to retain, the Eigenvalues above 1.0 criterion was applied following the evaluation of scree plot results [33,34]. Subsequently, the pattern matrix was examined to identify the loading and the structure of measured items on each factor. A measured item with low communality and substantial cross-loading between factors was removed from EFA. If cross-loading exists, the primary loading must be at least 0.20 larger than the second loading [33]. Further, to achieve satisfactory convergent validity of the factor solution for our sample size, each measured item load should be above 0.55 [35]. The discriminant validity of the EFA was evaluated by assessing the factors’ correlation matrix. Values < 0.70 indicate that factors are distracted from one another, providing unique information. Moreover, Cronbach’s alpha was applied to evaluate the reliability of each factor. Values above 0.7 indicate a highly reliable factor [33]. Additionally, factors should be theoretically meaningful [33]. Note that collinearity was also checked using the Variance Inflation Factor (VIF) test, where values were <3, showing no potential issues.

## 3. Results

The main descriptive statistics of the measured variables are presented in Table 2. These variables were analyzed with EFA to identify their associations and the best factor model that better interprets their underlying structure. The main results of the EFA are presented below.

**Figure 2:**
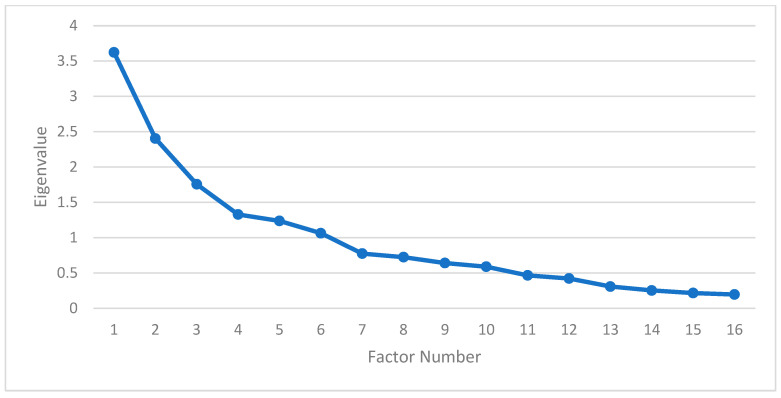
Scree plot that presents the eigenvalue for each factor.

EFA showed a middling KMO value of 0.680 and a statistically significant Bartlett’s test of sphericity < 0.001, which indicates the adequacy of the data for the analysis. Based on the EFA, the sixteen measured variables were reduced into six latent main factors, explaining 55.66% of the total variance. The main factors were chosen using the eigenvalue above one criterion as shown in the scree plot of Figure 2. The resulting six-factor model grouping the main measurement variables comprises hamstring (HS) and core endurance, HS strength, previous injuries, ABQ, lower limb (LL) strength, and strength limb symmetry (LS) (Table 3). In addition, Table 4 presents the communities of the measured items and the reliability values of the extracted latent factors.

Note that seven measurement variables were dropped from the EFA model. Particularly, SLR and SLR symmetry showed a negative correlation (−0.307). However, these measured items were heavily cross-loading with other strength and endurance measures and consequently were excluded from the final model. In addition, quadriceps LS, abductors LS, HS strength eccentric LS, SLHB LS, and the Biering–Sørensen test were also discarded from the final EFA model due to cross-loading. The above-mentioned LS variables exhibited low correlation values with various measures (all < 0.30). On the other hand, the Biering–Sørensen test showed a moderate positive correlation with endurance measures such as the prone bridge test (0.404), and lower correlation values with strength measures and asymmetries that result in cross-loading among endurance, strength measures, and asymmetries.

The latent factors’ correlation matrix is presented in Table 5, showing that each factor was distinct. Factors of previous injuries and ABQ provide a moderate correlation (0.335), and a low to moderate correlation is provided among HS and core endurance, LL strength, HS strength, and strength LS.

## 4. Discussion

### 4.1. Variables Interrelationships

As shown in this paper, the highly complex task of assessing the risk of injury can be simplified by consolidating the main variables related to HI into a parsimonious model of essential factors. The proposed model can practically assess the risk of injury, leading to an effective field-based screening protocol that adequately represents the necessary information from a larger set of variables. Specifically, the resulting EFA model suggests that sixteen measured items can be categorized into the six main factors of HS and core muscle endurance, HS strength, previous injuries, ABQ, LL strength, and strength LS.

The proposed six-factor model could guide the application of practical methods, reducing the otherwise time-consuming field-based evaluation for HI risk by excluding redundant measurements and applying only the most representative measures. Regarding that, although the baseline preseason evaluation needs to be as detailed as possible, in-season evaluation is also essential to capture possible dynamic modifications in the athlete that make him susceptible to injury [14,15]. For in-season evaluation to be practical and non-time-consuming, it is necessary to focus on the most representative higher-order parameters that better represent the athlete’s condition [13,14]. Then, sports scientists can focus on the specific possible impairments of athletes for a more detailed assessment that can guide the exercise-based prevention programs.

Precisely, the factors of HS and core endurance provide the most-explained variance according to the EFA. Core stability during high-speed running [10,11,36,37] and hamstring endurance [28] have been previously associated with HI. The evaluation of core stability in previous studies is based on three-dimensional laboratory measures and electromyographic core muscle activation patterns during high-speed running [10,11]. However, such evaluations are difficult to employ in field-based situations. The core endurance tests applied in the current investigation can provide an alternative and applicable method to evaluate athletes’ core stability and have recently been proven to have an association with HIs [38]. In addition, previous investigations [39], have provided evidence that impairments of core stability, as examined with measures similar to our study, have been associated with a higher likelihood of overuse injuries in athletes. Moreover, muscle fatigue is characterized as a key factor that may affect the high-speed running biomechanics, exposing the hamstrings to an increased risk of injury [37]. Regarding that, a decline in running ability during the final minutes of football matches has been identified [40]. Accordingly, various exercise protocols have been applied to enhance the running ability of football players, particularly during the final minutes of matches [41]. Consequently, incorporating HS and core endurance measures in the evaluation protocol is critical.

Further, while the Biering–Sørensen test constitutes a core endurance measure, it was excluded from the EFA as it provides associations with core endurance measures, strength measures, and asymmetries, resulting in cross-loading. Recently, it has been proposed that better performance in the Biering–Sørensen test has been associated with a lower risk of hamstring injuries in a cohort of rugby players [42]. The effect of the Biering–Sørensen test on other strength and endurance abilities should be further investigated [18]. Therefore, due to the high interrelationships of measurements, we could conclude that sports scientists can prioritize applying the prone bridge test for the evaluation of abdominal endurance and the SLHB test for hamstring muscle endurance. Consequently, the bilateral side bridge and Beiring–Sørensen test could be excluded from the in-season assessment and could be applied only if needed, for a more detailed evaluation.

Alternatively, strength measures were divided into two factors, one including the two isometric hamstring strength measures (make and brake test), and the other factor named LL strength, including the isometric strength of the quadriceps, abductors, and THD test. The above-mentioned separated grouping of strength measures was obvious due to the high intercorrelation between the two HS isometric measures. On the other hand, the THD test has previously been characterized as a measure to record LL strength and power [26], something that is approved by the grouping of lower limb strength measures in the EFA of the current study. According to that, the THD test could be characterized as a higher-order variable that incorporates the separated LL muscle strength abilities. In addition, while high-speed running biomechanics is crucial to be evaluated due to its connection with the HI mechanism [5], THD can be considered as an alternative method to simulate hamstring eccentric function when the evaluation of high-speed running is impossible to apply. Consequently, from a practical point of view, a brief screening protocol could be focused initially on the evaluation of the hamstring isometric strength using the “make” test and the THD test. Physical therapists need to consider that although the hamstring isometric “brake” test tends to simulate the eccentric hamstring strength that is an essential measure for hamstring injury risk, the “make” test is easier for athletes to understand, possibly providing more reliable results. Moreover, asymmetries of isometric hamstring strength (make test) and THD have moderate intercorrelation as they are loaded in the same factor and are important to consider in the evaluation protocol.

In addition, hamstring flexibility, although it constitutes a frequently measured variable in the screening of HI risk, was not approved by the literature to have an association with HI [7]. In the current investigation, hamstring flexibility provided a negative association with flexibility asymmetries, proving that higher flexibility is associated with decreased flexibility asymmetries. Flexibility asymmetries showed low association with factors like hamstring isometric LS and core stability measures and were excluded from the final EFA due to cross-loading. However, higher HS flexibility has been associated with lower hamstring peak strain at the late swing phase of high-speed running [43]. Considering that evidence, flexibility measures are important to incorporate in to the in-season evaluations for athletes who have been observed to have low flexibility or high asymmetries in the preseason evaluation.

Previous injuries are significant information regarding hamstring injury risk evaluation, as previous hamstring injuries during the same season are essential risk factors for subsequent HI [7]. Moreover, psychological measures such as athlete burnout have shown an association with hamstring injuries [12] and have been associated with previous injuries [38]. Using the ABQ can provide information on athletes’ sense of burnout that may be modified during the season and expose athletes to an increased risk of HI [12,44]. As hypothesized, measured indicators of these two factors were loaded into the different factors. However, as previously proposed by previous studies [12,44], we observed a moderate correlation between previous injuries and workload, suggesting that athletes with previous injuries may experience higher burnout. This relationship warrants further investigation to enhance our understanding of this pattern [12,44].

### 4.2. Practical Implications

Injury risk screening is an ongoing attempt and not a static condition [13,14,15]. Injury risk screening should include recursive loops of evaluation, application of exercise prevention strategies, re-evaluation, and possible individualized modifications to preventive strategies [45]. While preseason evaluation should be more detailed, in-season evaluation could focus on collecting more critical information, as proposed by the results of EFA, regarding previous injuries, athlete’s sense of burnout, abdominal endurance (prone bridge test), hamstring endurance (SLHB), hamstring isometric strength (make test), THD, asymmetries of hamstring isometric strength, and THD tests. This is a proposed six-factor model and does not exclude the possibility of adding additional measures during the in-season evaluation, based on the individualized impairments observed in athletes in preseason screening. For instance, as we proposed the prone bridge test for a representative measure of core stability, there is the possibility of adding the lateral bridge tests in cases that have observed high asymmetries in lateral abdominus endurance during the preseason evaluation. Signals such as lower ability in measurements, increased asymmetries due to microinjuries or fatigue, and increased burnout measured with ABQ, during the in-season evaluation, may provide evidence that these athletes should be protected by applied specific individualized prevention strategies [13]. The time scale of the evaluation during the season cannot be proposed by the results of the study. A previous paper proposed that the evaluation of the athletes should be on a daily or weekly basis in order to capture fluctuations in athletes’ abilities and increase the possibility of injury forecasting [14]. We proposed that the current evaluation protocol, to be practical, should be applied at least on a monthly or bimonthly time scale. However, future research should evaluate this issue and provide more quantitative results.

**Figure 3:**
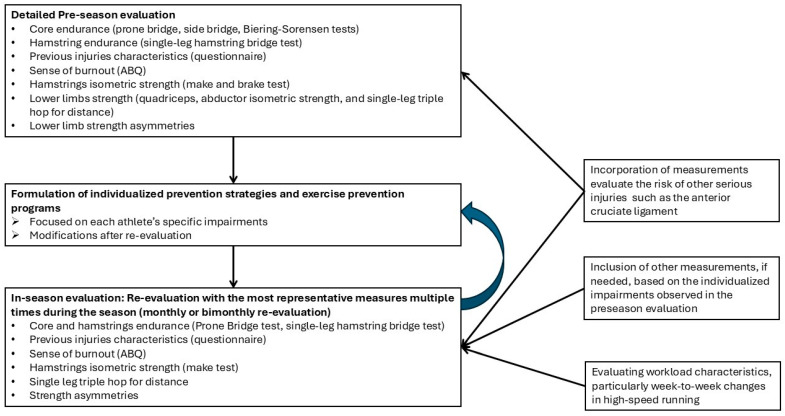
A proposed framework for the practical application of the field-based screening protocol.

Further, the proposed protocol should be combined with frequent workload screening, as workload characteristics such as week-to-week changes in high-speed running exposure have been associated with HI [9]. In addition, as HI presents among the highest incidence in football players, this protocol must be combined with measurements evaluating the risk factors for other serious injuries, such as anterior cruciate ligament tears [18]. Figure 3 provides a framework for the practical application of the proposed protocol.

### 4.3. Limitations and Future Research Directions

Despite the valuable results of this study, several limitations should be considered. Firstly, a larger sample size could provide more validity in the results, particularly regarding the measures that were excluded from the EFA, such as HS flexibility, asymmetries, and the Biering–Sørensen test. A simple guideline for the use of EFA regarding the sample size is at least 100–200 observations, and the 99 participants of our study are slightly lower than this proposed threshold. Additionally, other important measures, such as the high-speed running qualitative assessment [46] were not included in our study. Moreover, as this study is cross-sectional, a prospective cohort study is important to assess the ability of the evaluation protocol to detect increased risk for HI. Another possible limitation is that the results are representative of male professionals and semiprofessional football players, and cannot be generalized to other populations, such as recreational, women, or other sports athletes. Finally, while the proposed evaluation protocol requires low-cost equipment, with the most expensive being the hand-held dynamometer, costing approximately 500–1000 $, it remains essential to provide specialized training for examiners to ensure the accurate application of the measurements.

Note that the resulting six-factor field-based protocol model could constitute the basis for future research and the development of practical methods in assessing the risk and thereby preventing injuries. Particularly in investigations with higher sample sizes, incorporating other critical measures, such as the qualitative assessment of high-speed running in various samples, would provide valuable information. Finally, prospective investigations would provide information on the ability of this protocol to detect athletes who are at risk of injury.

## 5. Conclusions

HI risk screening protocols should be valid but at the same time practical for effective application by sports scientists. There are multiple measures that can be utilized to evaluate the risk of HI in football players, leading to high complexity in the assessment. Risk screening protocols should provide the most important information required to capture the modifications on athletes’ injury risk profiles by applying the screening multiple times during the season [14,15]. The application of EFA reduces the twenty-three measured variables into a parsimonious six-factor model, including core muscle endurance, hamstring isometric strength, previous injuries, athlete’s sense of burnout, lower limb strength, and asymmetries of lower limb strength. The resulting model provides a practical protocol, facilitating sports scientists in evaluating the risk for HI in the highly complex reality of field-based situations.

## Figures and Tables

**Table 1:** Participants’ Characteristics.

	Professional (*n* = 52) (Mean ± SD)	Semi-Professional (*n* = 47) (Mean ± SD)	Total (*n* = 99) (Mean ± SD)
Age	22.50 ± 4.53	21.79 ± 5.55	22.16 ± 5.03
Weight (kg)	75.42 ± 7.77	73.36 ± 7.58	74.44 ± 7.71
Height (cm)	179.89 ± 6.65	177.64 ± 5.67	178.82 ± 6.27
BMI	23.27 ± 1.69	23.22 ± 1.85	23.25 ± 1.76
Football Start Age	7.77 ± 2.77	7.72 ± 3.14	7.75 ± 2.94
Years Playing at Professional Level	3.87 ± 4.05	3.70 ± 4.11	3.79 ± 4.06
Games Participation Previous Year	20.27 ± 11.14	18.15 ± 7.17	19.26 ± 9.48
Hours of Training per Day in the Previous Year	2.40 ± 0.63	2.24 ± 0.65	2.33 ± 0.64
Days Training per Week in the Previous Year	5.76 ± 0.43	5.27 ± 0.66	5.53 ± 0.60

**Table 2:** Descriptive statistics of the measured variables.

Variables Category	Variables (Unit of Measurement)	Mean	SD	Skewness		Kurtosis	
				Statistic	Std. Error	Statistic	Std. Error
Previous Injuries	Number of Previous Injuries	0.90	0.83	0.52	0.24	−0.51	0.48
	Time Loss of the Most Recent Injury (5-linked scale, 0 = no injury, 4 = greater than 3 months’ time loss)	1.47	1.34	0.29	0.24	−1.22	0.48
ABQ	Emotional Physical Exhaustion (5-linked scale)	1.65	0.48	0.89	0.24	0.48	0.48
	Reduced Sense of Accomplishment (5-linked scale)	2.45	0.56	−0.37	0.24	0.39	0.48
	Devaluation (5-linked scale)	1.34	0.63	2.67	0.24	7.84	0.48
LL Neuromuscular Characteristics	SLR (°)	77.18	8.66	−0.03	0.24	−0.14	0.48
	Strength Abductors (Nm/kg)	2.28	0.28	0.00	0.24	−0.44	0.48
	Strength HS (brake test) (Nm/kg)	1.61	0.24	0.53	0.24	0.34	0.48
	Strength HS (make test) (Nm/kg)	1.49	0.21	0.47	0.24	0.98	0.48
	Strength Quadriceps (Nm/kg)	3.04	0.46	0.01	0.24	−0.18	0.48
	THD (cm/body height)	3.23	0.31	0.28	0.24	0.97	0.48
	SLHB (maximum repetitions)	32.77	9.99	0.13	0.24	−0.10	0.48
LS	Strength Abductors LS (%)	8.12	6.33	0.94	0.24	1.47	0.48
	Strength HS (brake test) LS (%)	6.90	5.16	0.68	0.24	−0.30	0.48
	Strength HS (make test) LS (%)	5.65	4.43	1.10	0.24	1.17	0.48
	Strength Quadriceps LS (%)	7.30	6.01	1.12	0.24	0.69	0.48
	SLR LS (%)	5.55	4.22	1.08	0.24	0.52	0.48
	SLHB LS (%)	13.68	10.42	0.55	0.24	−0.55	0.48
	THD LS (%)	4.90	4.04	1.07	0.24	0.90	0.48
Core Endurance	Prone Bridge (seconds)	175.40	76.12	0.84	0.24	0.11	0.48
	Side Bridge D (seconds)	90.10	28.71	0.98	0.24	1.03	0.48
	Side Bridge ND (seconds)	89.61	27.73	0.57	0.24	−0.55	0.48
	Biering–Sørensen Test (seconds)	100.30	36.95	0.57	0.24	0.33	0.48

Abbreviations: ABQ—Athlete Burnout Questionnaire, HS—Hamstring, SD—standard deviation, LL—Lower Limbs, SLR—Single Leg Raise, THD—Triple Hop Distance, SLHB—Single Leg Hamstring Bridge, ND—Non-Dominant.

**Table 3:** EFA pattern matrix of variables grouping into latent factors. Only coefficients > 0.2 are presented.

Measured Items	Factor (% of Explained Variance)					
	F1 (20.24%) HS and Core Endurance	F2 (12.81%) HS Strength	F3 (8.93%) Previous Injuries	F4 (5.75%) ABQ	F5 (4.10%) LL Strength	F6 (3.79%) Strength LS
Side Bridge D	0.873					
Side Bridge ND	0.865					
Prone Bridge	0.731				0.215	
SLHB D	0.558	0.246				
Strength HS (brake test)		0.925				
Strength HS (make test)		0.727				
Number of Previous Injuries			0.994			
Time Loss of the Most Recent Injury			0.706			
Emotional Physical Exhaustion				0.866		
Devaluation				0.624		
Reduced Sense of Accomplishment				0.454		
Strength Quadriceps					0.647	
THD					0.427	−0.297
Strength Abductors				0.230	0.419	
THD LS						0.672
Strength HS (make test) LS	−0.200					0.442

Abbreviations: ABQ—Athlete Burnout Questionnaire, HS—Hamstrings, SLR—Single Leg Raise, THD—Triple Hop Distance, SLHB—Single Leg Hamstring Bridge. The background color clarifies the grouping of the variables into the latent factors according to their loading values.

**Table 4:** Measured items’ communities and latent factors’ reliability values.

Measured Items	Communalities	Extracted Factor	Cronbach’s Alpha
Side Bridge D	0.750	HS and core Endurance	0.68
Side Bridge ND	0.743		
Prone Bridge	0.652		
SLHB D	0.377		
Strength HS (brake test)	0.942	HS Strength	0.835
Strength HS (make test)	0.563		
Number of Previous Injuries	0.908	Previous Injuries	0.767
Time Loss of the Most Recent Injury	0.606		
Emotional Physical Exhaustion	0.754	ABQ	0.655
Devaluation	0.365		
Reduced Sense of Accomplishment	0.327		
Strength Quadriceps	0.451	LL Strength	0.525
THD	0.343		
Strength Abductors	0.345		
THD LS	0.445	Strength LS	0.489
Strength HS (make test) LS	0.336		

Abbreviations: ABQ—Athlete Burnout Questionnaire, HS—Hamstring, THD—Triple Hop Distance, SLHB—Single Leg Hamstring Bridge.

**Table 5:** Latent factors’ correlation matrix. The last row explains the strength of positive (blue) and negative (red) correlations.

Factor							HS and Core Endurance			HS Strength				Previous Injuries			ABQ			LL Strength			Strength LS		
HS and Core Endurance							1.000			0.283				−0.087			0.139			0.288			−0.297		
HS Strength										1.000				0.173			0.065			0.323			−0.216		
Previous Injuries														1.000			0.335			0.023			0.029		
ABQ																	1.000			0.121			0.006		
LL Strength																				1.000			−0.156		
Strength LS																							1.000		
1	0.9	0.8	0.7	0.6	0.5	0.4		0.3	0.2		0.1	0	−0.1		−0.2	−0.3		−0.4	−0.5	−0.6	−0.7	−0.8		−0.9	−1

## Data Availability

The data supporting the findings of this study are available on reasonable request from the corresponding author. The data are not publicly available due to privacy and ethical restrictions.
